# Supplementary material for: Procalcitonin Evaluation of Antibiotic Use in COVID-19 Hospitalised Patients (PEACH): Protocol for a Retrospective Observational Study
Source: Methods Protoc. 2022 Nov 28;5(6):95. doi: 10.3390/mps5060095 (PMC9786133; doi:10.3390/mps5060095)
Supplement: Supplementary file 1 [file mps-05-00095-s001.zip › mps-1985998-SI/statisical Analysis Plan.pdf]

Statistical Analysis Plan for PEACH: Procalcitonin Evaluation of Antibiotic Use in COVID-19 Hospitalised Patients<br><br>Work Package 2.1 – Patient-level impact of PCT testing on antibiotic exposure and clinical outcome			
Eudract/ISRCTN No:	ISRCTN66682918<br><br>(https://www.isrctn.com/ISRCTN66682918)	Version Number:	1.0 (07/09/2022)

**Draft Plan**
Based on protocol version: 1.2 (25/08/2022)<br><br>PEACH Protocol Version V1.2 25.08.22.docx

SAP Revision History				
Protocol version	Updated SAP version no.	Section number changed	Description and reason for change	Date changed

ROLES AND RESPONSIBILITIES

**Study Statisticians:** Dr Detelina Grozeva, Dr Edward Webb (Health Economics)			
**Role:**			
Date:		Signature:	
**Senior Statisticians:** Dr Philip Pallmann, Prof Robert West, Assoc Prof Bethany Shinkins (Health Economics)			
**Role:**			
Date:		Signature:	
**Chief Investigator:** Dr Jonathan Sandoe (CI), Prof Enitan Carrol (co-CI)			
**Role:**			
Date:		Signature:	
**Other non-signatory contributors to the SAP:** n/a			
**Role:**			

1. BACKGROUND

1.1 RATIONALE AND RESEARCH QUESTION

See protocol.

1.2 OBJECTIVES

The main aim is to measure the difference in early antibiotic use between COVID-19 patients who did/did not have procalcitonin testing (PCT) at the time of COVID-19 diagnosis.

Secondary objectives include:

- To measure the difference in antibiotic use (total and 'late'), length of stay (in hospital, post COVID-19 and ICU), mortality (30- and 60-day), intensive care unit admission, and resistant bacterial infections ('superbugs') between COVID-19 patients who did/did not have PCT testing at baseline or PCT at any time during therapy
- To assess the relationship between the number of PCT tests and total days of antibiotic therapy
- To assess the relationship between C-reactive protein (CRP) and PCT to test if a low CRP predicts a low PCT
- To describe the relationship between PCT and other inflammatory markers (neutrophil count, white cell count)
- To assess the relationship between tocilizumab and dexamethasone on inflammatory markers including PCT.

2. STUDY MATERIALS

2.1 STUDY DESIGN

Multicentre, retrospective observational cohort study using patient-level clinical data. The data are collected in 11 NHS acute hospital Trusts and Health Boards in England and Wales. The study has been designed with consideration of STROBE criteria and intending these criteria to be followed for reporting (von Elm E *et al.,* 2007).

2.2 RANDOMISATION

Not applicable.

To account for potential systematic differences in baseline characteristics between the patients who had PCT testing and those who did not, propensity score matching (or similar covariate balancing techniques) will be used.

2.3 SAMPLE SIZE

Data from ~7,000 COVID-19 patients will be available from 11 NHS acute hospital Trusts and Health Boards from England and Wales with around half having had PCT testing, while the other half non-PCT-tested patients. Based on a minimally important clinical difference in antibiotic duration of one day (ref ADAPT-Sepsis trial protocol: https://warwick.ac.uk/fac/sci/med/research/ctu/trials/adaptsepsis/3.2_adapt-sepsis_trial_protocol__v3.0_12-feb-2019_clean.pdf) between baseline PCT and non-PCT-tested patients, and a conservative assumption for the standard deviation (SD) of six days, a sample set of 1,500 matched patients was estimated to provide 90% power when using a two-sided test with 5% alpha to detect a difference of one day of antibiotic therapy.

2.4 FRAMEWORK

Retrospective analysis of routinely collected data by 11 NHS acute hospital Trusts and Health Boards from England and Wales, some of which did/did not use PCT routinely in COVID-19 patients.

2.5 INTERIM ANALYSES

2.5.1 PLANNED SAMPLE SIZE ADJUSTMENT

Not applicable.

2.5.2 STOPPING RULES

Not applicable.

2.6 TIMING OF FINAL ANALYSIS

Once all data have been made available by our partners and captured in a MACRO database.

2.7 TIMING OF OUTCOME ASSESSMENT

The data will be inputted into a MACRO database by our partners covering the following period: patients admitted with COVID-19 between 01/02/2020 and 30/06/2020. This period encompasses the first peak of the COVID-19 pandemic across the UK.

3. STATISTICAL PRINCIPLES

3.1 LEVELS OF CONFIDENCE AND P-VALUES

All hypothesis tests and confidence intervals (CIs) will be two-sided. We will use a 5% significance level and present 95% CIs.

3.1.1 ADJUSTMENT FOR MULTIPLICITY

Not applicable.

3.2 ADHERENCE AND PROTOCOL DEVIATIONS

3.2.1 DEFINITION AND ASSESSMENT OF ADHERENCE

Not applicable.

3.2.2 PRESENTATION OF ADHERENCE

Not applicable.

3.2.3 DEFINITION OF PROTOCOL DEVIATION

Not applicable.

3.2.4 PRESENTATION OF PROTOCOL DEVIATIONS

Not applicable.

3.3 ANALYSIS POPULATION

Data from all patients ≥16 years admitted to a participating NHS hospital and with COVID-19 between 01/02/2020 and 30/06/2020 are eligible. All effort will be made to collect data from all eligible patients for the studied period, although potentially this may not be feasible. We expect that around 10% of patients' data may prove impossible to collect.

Participants will be identified from institutional databases by the clinical teams and anonymised data will be entered into the study MACRO database.

To reduce the risk of bias, consecutive patients fulfilling the eligibility criteria will be included. This will be facilitated by the use of retrospective collection of prospectively collected institutional clinical data.

4. STUDY POPULATION

4.1 SCREENING DATA

Not applicable.

4.2 ELIGIBILITY

Confirmed COVID-19 (positive PCR test) and admitted to participating NHS Trusts/hospitals (for any reason) for the study period: 01/02/2020 to 30/06/2020. The age limit is ≥16 years.

Second and subsequent admissions after the index admission with COVID-19 will be excluded.

4.3 RECRUITMENT

Not applicable.

4.4 WITHDRAWAL/FOLLOW UP

4.4.1 LEVEL OF WITHDRAWAL

Not applicable.

4.4.2 TIMING OF WITHDRAWAL

Not applicable.

4.4.3 REASONS FOR WITHDRAWAL

Not applicable.

4.4.4 PRESENTATION OF WITHDRAWAL/LOSS TO FOLLOW-UP

If patients are excluded from analysis, the corresponding numbers and reasons for exclusion will be listed in a table and/or presented by arm in a flow diagram.

4.5 BASELINE PARTICIPANT CHARACTERISTICS

4.5.1 LIST OF BASELINE DATA

Routinely collected data from a patient's episode of COVID-19 will be collected, in a standardised format into a dedicated study database (MACRO). The data will be collected from institutional clinical databases and patient medical records.

'Baseline' data include:

- Site
- Patient demographics (age, sex, ethnicity)
- Comorbidities (Quality Outcome Framework registered conditions, frailty scores)
- Lower-level super output area (LSOA, to allow derivation of index of multiple deprivation, IMD)
- Body mass index (BMI)
- Smoking status
- Penicillin allergy or sensitivity status
- Date of hospital admission/discharge/death (to derive length of hospital stay, survival time and 30 and 60-days mortality rates)
- Date of admission/discharge to intensive care unit (to derive length of hospital stay in ICU, ICU admission status)
- Antibiotics used during COVID-19 episode (agent, dose, route, start and stop dates; these data will be used to derive days and defined daily doses (DDDs), of 'early', 'late' and total antibiotic treatment)
- Antivirals used
- Resuscitation status and level of care preference
- Lung imaging findings (at baseline and during follow-up). Imaging variables include: COVID-19 categorisation of imaging at baseline; and presence of new consolidation subsequently during the episode
- Physiological observations at time of diagnosis (respiratory rate, systolic blood pressure, consciousness, Glasgow coma score, temperature, Oxygen saturation, qSOFA, NEWS2, CURB-65 scores, 4C Mortality Score for COVID-19)
- Laboratory tests: Positive COVID-19 test date, PCT test results during the episode and dates, and baseline: urea, creatinine, C-reactive protein, troponin, ferritin, D-dimer, white cell count, lymphocyte and neutrophil counts, haemoglobin and platelets
- Microbiology results and date of sampling for: blood cultures, respiratory samples and Clostridium difficile testing.

4.5.2 DESCRIPTIVE STATISTICS

Descriptive (e.g. means or medians with standard deviations or interquartile ranges for continuous variables, frequencies and percentages for binary or categorical variables) and graphical summaries (e.g. boxplots, histograms, etc.) will be presented by exposure to baseline PCT testing (yes or no).

The descriptive variables will be demographics, underlying condition, BMI, penicillin allergy status, length of hospital stay (from admission and from date of COVID-19 diagnosis), length of ICU admission stay, number of deaths (30- and 60-days), baseline acute physiology, baseline acute kidney injury (AKI), baseline laboratory values, baseline positive/negative PCT (cut off values for a positive result: above 0.25 and above 0.50 ug/L), duration of early/late/total antibiotic use, antiviral use, types of antibiotic, the relationship between different inflammatory markers and outcomes, route of administration, frequency of PCT testing, types of secondary bacterial infection amongst other variables listed in 4.5.1 above.

Additional descriptive statistics will be provided per NHS trust with respect to the corresponding hospital policy for the implementation of PCT usage (yes/no), where PCT was used (ICU or locations outside of ICU), specific PCT cut off values for stopping or withholding antibiotics (0.25 or 0.50 ug/L) and did the hospital adopt/expand PCT testing in the 1st wave of the COVID-19 pandemic.

Number of PCT tests per person and PCT usage in ICU and non-ICU hospital locations will be also presented.

Similar descriptive statistics as the ones described above for PCT, will be presented for CRP (cut off value for a positive result: >10 mg/L) and for neutrophil (cut off value >7x 10*9/L).

Missingness per variable and by group will be reported.

5. ANALYSIS

5.1 OUTCOME DEFINITIONS

5.1.1 PRIMARY OUTCOME(S)

Primary outcome is days of early antibiotics therapy (within the first seven days after a first positive COVID-19 test sample).

5.1.2 TIMING, UNITS AND DERIVATION OF PRIMARY

Day 1 of COVID-19 is considered the day of the first positive COVID-19 test sample. 'Early' antibiotic use is considered prescriptions between days 1 to 7, and 'late' antibiotic use - prescriptions after day 7. The count for the corresponding number of days on antibiotic treatment starts on the 1st day of the prescription (that is, the period includes both 1st and last day).

5.1.3 LIST OF SECONDARY OUTCOMES

- Total duration/length of antibiotic treatment (days therapy)
- Duration (days) of late (>7 day) antibiotic treatment
- Total DDDs of antibiotics
- DDDs of early (< 7 days) antibiotic treatment
- DDDs of late (>7 day) antibiotic treatment
- Appropriateness of antibiotics according to local guidelines (% compliance), if practicable
- 30-day mortality
- 60-day mortality
- ICU admission
- ICU length of stay
- Length of hospital stay (total and from day 1 post COVID-19 positive test)
- Antimicrobial resistant (late onset) secondary bacterial infection.

5.1.4 ORDER OF TESTING

Not applicable.

5.1.5 TIMING, UNITS AND DERIVATION OF SECONDARIES

Total duration of antibiotic use is all prescriptions from day 1 to date of death or discharge (that is, includes both 1st and last day).

Hospital-onset COVID-19 is defined as: 'probable', first COVID-19 positive sample 8-14 days after admission; 'definite', first COVID-19 positive sample >15 days after admission positive COVID-19 swab prior to day 8 of admission is considered to be either Community-Onset (day 0-2) or Indeterminate (day 3-7) (NHS England: https://assets.publishing.service.gov.uk/government/uploads/system/uploads/attachment_data/file/961210/S1056_Contribution_of_nosocomial_infections_to_the_first_wave.pdf).
'Early secondary bacterial infection' is considered to be present if there is a significant positive blood cultures or deep respiratory samples, collected on or up to two days after diagnosis of COVID-19 i.e., diagnosed days 1-3. Early secondary bacterial infection is not an outcome measure but is considered as a factor potentially affecting antibiotic prescribing and propensity to procalcitonin testing.

Two definitions of 'late onset secondary bacterial infection' are used to capture the uncertainty around diagnosis of secondary bacterial infection and assessed in sensitivity analyses: i) A culture-based specific but insensitive definition is considered as: significant positive blood cultures or deep respiratory samples, collected >48h after day 1 i.e. from day 4 onwards; ii) A clinically-based sensitive but non-specific definition is considered as clinician commencing a new antibacterial prescription >48h after day 1 i.e. from day 4 onwards. For the purposes of this study 'late onset secondary infections' is considered a description variable and 'Resistant late onset secondary infections' are considered an outcome measure.

Resistant late onset secondary bacterial infections are defined as resistant to three or more antibiotic classes (with sensitivity analyses for resistance to $\geq$1 or >2 classes). Blood cultures are considered to be positive if they grow a recognised pathogen from at least one set of cultures, or if they grow an organism known to contaminate blood cultures (such as coagulase negative staphylococci and other skin commensals) from at least two sets. Deep respiratory cultures are considered to be positive if they grow a recognised pathogen. Microbiology samples are all processed in United Kingdom Accreditation Service (UKAS) accredited laboratories according to local standard operating procedures.

The findings of chest x-rays and computer tomography are collected as per radiology or clinician reports and categorised as per the British Society of Thoracic Imaging guidance (Hare SS, Rodrigues JCL, Nair A, et al. The continuing evolution of COVID-19 imaging pathways in the UK: a British Society of Thoracic Imaging expert reference group update. Clin Radiol 2020; 75(6): 399-404.): CVCX0 = normal, CVCX1 = classic/probable COVID-19 infection (lower lobe and peripheral predominant multiple opacities that are bilateral), CVCX2 = Indeterminate (does not fit classic or non-COVID-19 descriptors) CVCX3 = Non COVID-19 (Lobar pneumonia, pleural effusions, pulmonary oedema or other patterns).

Acute kidney injury (AKI) is measured on day 1 (yes/no) along with the AKI stage on day 1 worst value (Stage 1 to 3).

5.2 ANALYSIS METHODS

5.2.1 LIST OF METHODS AND PRESENTATION

- **Descriptive statistics** will be used for rates of PCT testing, antibiotic prescribing, and secondary bacterial infection. This will be done overall and separately for those hospitals using/not using PCT, and also separately for patients who did/did not receive a baseline PCT test and a PCT test at any time. Antibiotic prescribing will be described as 'early' (within first 7 days of COVID-19 diagnosis), 'late' (more than 7 days), and total use ('early' plus 'late'), and broken down by type of antibiotic (e.g., broad- or narrow-spectrum) and by hospital.
- To assess the effect of PCT testing on antibiotic prescribing and patient outcomes and to ensure an even distribution of confounders between groups, **propensity score matching** will be used. We will estimate a patient's propensity for PCT testing (outcome binary variable: "yes"/"no") with a logistic regression on patient characteristics including age, sex, BMI, comorbidity, smoking, ethnicity, index of multiple deprivation (IMD), baseline clinical severity of illness assessments (qSOFA, CURB-65, NEWS score, 4C mortality score for COVID-19), baseline AKI, early secondary bacterial infection, baseline lung imaging category, blood tests (e.g. baseline CRP, neutrophil count, white cell count, D-dimer, troponin). The covariates will be selected if they might affect choices about PCT testing. We will assess different ways of carrying out the propensity score matching: using a nearest-neighbor matching algorithm without a caliper/with a caliper or propensity score weighting (Nguyen TL *et al.*, 2017; Li F *et al.*, 2018; Markoulidakis A *et al.,* 2021;). In addition, we will estimate the

propensity score using covariate balancing propensity score, which has been shown to lead to optimised resulting covariate balance. (R package CBPS, Imai K and Ratkovic M, 2013).

Patients who did or did not receive PCT testing will be matched (without replacement) with a 1:1 or 1:2 ratio according to their propensity. This will enable the comparison of several outcomes between-patient groups which are balanced on important known confounders. Potential hospital effects will be accounted for in the model (e.g., by using random effects) or will be absorbed into the propensity scores.

Balance diagnostics will be assessed to check if the propensity score model has been adequately specified. This will involve examining if the distribution of measured baseline covariates is similar between patients who received the PCT test and who did not. Comparing the similarity in the matched sample will be done by comparing of the means or medians for continuous covariates and the distribution of the categorical variables between patients who received the PCT test and who did not. Kolmogorov-Smirnov statistics has been shown to capture the whole distribution well and will be used to assess the balance on the baseline covariates (with 0.1 shown to be a suitable threshold to consider as acceptable balance) (Markoulidakis A *et al.,* 2021). The standardised mean difference (SMD) will also be used to assess the balance. SMD of <0.1 has been suggested to indicate a negligible difference in the mean or prevalence of a covariate. If SMD > 0.1, double-adjustment on propensity score-matched samples has been shown to address the issue of residual confounding bias (Nguyen TL *et al.*, 2017).

To further assess the propensity score matching model, descriptive tables of matched/unmatched samples for the covariates of interest will be presented. Graphical diagnostics will also be used to assess group balance for the covariates after propensity score matching, such as boxplots, quantile-quantile plots, and nonparametric density plots.

In addition, alternative matching methods such as Mahalanobis distance matching, optimal matching and coarsened exact matching will be explored (Iacus SM *et al.*, 2017). If the propensity score matching model is found to be inadequate, other methods for propensity score analysis will be applied and further assessed. These methods include stratification (or subclassification) on the propensity score, inverse probability of treatment weighting using the propensity score, and covariate adjustment using the propensity score (Li F *et al.*, 2018).

Once an appropriate level of between-group balance of the confounders/covariates is achieved, the matched data set will be ready for the **main analysis** (using **Poisson regression**) of between-group difference in the study primary and secondary outcomes:

- **Multivariable regression models** with random hospital effects will be used to examine factors affecting antibiotic prescribing. The variables used in the analysis of **early, late and total antibiotic prescribing** are presented in the following table for clarity:

Variables to include in the multivariable regression models	Early antibiotic use	Late antibiotic use	Total antibiotic use
age	✓	✓	✓
sex	✓	✓	✓
ethnicity	✓	✓	✓
smoking status	✓	✓	✓
comorbidity	✓	✓	✓
IMD	✓	✓	✓
penicillin allergy status	✓	✓	✓
baseline lung imaging category	✓		✓
most recent lung imaging category		✓	✓
presence/absence early secondary bacterial infection	✓		✓
presence/absence most recent secondary bacterial infection		✓	✓
late secondary bacterial infection		✓	✓
baseline PCT levels	✓		✓
most recent PCT levels		✓	
baseline CRP	✓		✓
most recent CRP		✓	
baseline white cell count	✓	✓	✓
baseline neutrophil count	✓	✓	✓
baseline severity of illness: 4Cs mortality score for COVID	✓*	✓*	✓
ICU admission	✓	✓	✓
remdesivir treatment		✓	✓
dexamethasone treatment		✓	✓
early antibiotic use and duration		✓	✓

*- we will also explore the use of CURB-65, qSOFA and NEWS in sensitivity analyses

- Results will be presented as effect estimates (the average treatment effect on the entire population (ATE, Markoulidakis A *et al.,* 2021)) with 95% CIs and p-values. Baseline PCT and PCT at any time will be assessed; it is hypothesised that a baseline PCT may influence duration of early antibiotic therapy while a PCT at any time may influence duration of total or late antibiotic therapy.
- The primary analysis model for the propensity score-matched data will depend on the type of outcome e.g. logistic regression for binary outcomes (e.g. ICU admission, antimicrobial resistant (late onset) secondary bacterial infection, 30-day/60-day mortality) and linear regression for continuous outcomes (e.g. days on antibiotics, total DDDs of antibiotics, DDDs of late (>7 day) antibiotic treatment, ICU length of stay, length of hospital stay, duration (days) of late (>7 day) antibiotic treatment).
- Secondary analysis: We will explore if the number of the performed PCT tests is associated with the duration of the antimicrobial treatment.

- Secondary analysis: The above will be adjusted for “truncation by death” i.e. the problem that some patients die before another outcome (e.g. days on antibiotics) can be fully measured, thus leaving these outcome measures censored/undefined and with a seemingly better outcome (e.g. fewer days on antibiotics) due to the early death. To take this into account, in addition to a crude analysis, restricted to the survivors in each group, **survivor average causal effect** (SACE) analysis of the “always-survivors” will also be performed, i.e. those who would have survived in either group (Tchetgen EJT, 2014).
- Secondary analysis: **Survival analysis** will also be undertaken for outcomes that could be expressed as time-to-event (e.g., time until antibiotics are stopped) adjusting for confounders using a Cox regression if the proportional odds assumption holds, and after stratification otherwise. This will provide greater power than the above analyses but will require further modelling assumptions. Importantly, it will allow us to fit competing risks modelling with death being a “competing risk”.

5.2.2 COVARIATE ADJUSTMENT

Described above

5.2.3 ASSUMPTION CHECKING

We will perform standard regression diagnostics (e.g. residuals vs. fitted plots) to assess basic assumptions such as linearity, normality, and homoscedasticity, as well as inspecting outliers.

5.2.4 ALTERNATIVE METHODS IF DISTRIBUTIONAL ASSUMPTIONS NOT MET

If the distributional assumption for Poisson distribution is not met, fitting models based on Quasi-Poisson or negative binomial distributions will be considered.

5.2.5 SENSITIVITY ANALYSES

- Where appropriate, sensitivity analyses, including multiple imputation, will be undertaken to explore the impact of missing data.
- Further sensitivity analysis will be performed to measure if there are other confounding factors that have not been collected in the dataset. The unmeasured confounding in the study will be assessed by using E-values (VanderWeele TJ and Ding P, 2017; Haneuse S *et al.*, 2019).
- The use of CURB-65, qSOFA and NEWS scores will be explored in the main analyses.
- The rates of PCT testing in different hospitals may be different; also, the testing rates at the end of the study period may be different than the rates at the beginning of the period. In order to account for these, we will perform the following sensitivity analysis: the propensity model to reflect hospital, or to group hospitals by rates of PCT and match within groups/strata.

- Patients who did or did not receive PCT testing will be matched without replacement with a 1:1 or 1:2 ratio according to their propensity for the main analysis. If a good balance is not achieved, matching with replacement will be performed as a sensitivity analysis.
- Resistant late onset secondary bacterial infections are defined as resistant to three or more antibiotic classes (in 5.1.5, page 8). Sensitivity analyses will be performed for resistance to >1 or >2 classes.

5.2.6 SUBGROUP ANALYSES

The main analysis will be repeated for the group of patients who were tested with PCT. The exposure (independent variable) in the multivariable models will be PCT positive/negative result.

5.3 MISSING DATA

- Patterns of missingness will be explored and summarised descriptively by PCT usage group.
- Where appropriate, sensitivity analyses, including multiple imputation, will be undertaken to explore the impact of missing data. Before performing multiple imputation, the Little's test will be used for assessing if data are missing completely at random (Little RJA, 1988).

5.4 ADDITIONAL ANALYSES

- To assess the relationship between CRP and PCT, an additional analysis will be performed (i.e. whether low CRP can predict low PCT, similar to Houghton R et al. C-reactive protein-guided use of procalcitonin in COVID-19, JAC, 2021, https://academic.oup.com/jacamr/article/3/4/dlab180/6445222). The correlation (Pearson) between the results obtained by the CRP and PCT tests for the same individuals will be presented, along with 2x2 contingency table. The correlation (Pearson) between baseline PCT and neutrophil count will be presented too.
- The PSM for propensity for CRP testing and neutrophil testing will be performed similarly to what will be done for the propensity to PCT testing. Subsequently, the main analysis will be performed too.
- The clinical outcomes by PCT result will be explored to test if high PCT predicts poor outcome.

5.5 HARMS

Not applicable.

5.6 STATISTICAL SOFTWARE

The analyses will be performed in R version 4.0.0 or higher, with add-on packages such as 'MatchIt' (Ho D *et al.*, 2011) for propensity score matching, 'EValue' (Mathur MB *et al.,* 2018) for unmeasured confounding, 'MatchThem' (Pishgar F *et al.*, 2021) for propensity score matching and weighting after multiple imputation, CBPS for estimation of the propensity score using covariate balancing propensity score (Imai K and Ratkovic M, 2013) and 'ggplot2'

for graphical presentation. STATA v17 will be used for data management and descriptive tables.

6. HEALTH ECONOMICS

In addition to the individual patient data analysis, the health economics analysis will include a decision analytic model (Briggs A et al., 2006), which will combine information about costs, clinical outcomes, and patients' health-related quality of life. The model will be populated with costs and outcomes obtained from the individual level data analysis. A systematic search of the literature will identify published health-related quality of life values for inpatients with COVID-19. The structure of the model will be partially informed by a scoping search of the published literature to identify existing models comparing PCT testing to current practice (likely majority of literature is focused on non-COVID 19 patient populations). The model is likely to consist of an initial decision tree to capture the comparative diagnostic pathways, with the possibility of using a Markov model to capture downstream health and cost consequences. Results will be presented as net health benefit and incremental cost-effectiveness ratios. Sensitivity analyses will explore how robust the model is to varying its underlying assumptions and uncertainty in parameter estimates.

7. REFERENCES

7.1 NON-STANDARD STATISTICAL METHODS

Von Elm E, *et al*. The Strengthening the Reporting of Observational Studies in Epidemiology (STROBE) statement: guidelines for reporting observational studies. PLoS medicine (2007)

Nguyen TL, *et al*. Double-adjustment in propensity score matching analysis: choosing a threshold for considering residual imbalance. BMC Med Res Methodol (2017). https://doi.org/10.1186/s12874-017-0338-0

Li F., *et al*. Balancing covariates via propensity score weighting. Journal of the American Statistical Association (2018)

Markoulidakis A, *et al*. A tutorial comparing different covariate balancing methods with an application evaluating the causal effects of substance use treatment programs for adolescents, arXiv (2021); https://arxiv.org/abs/2010.09563

Imai K and Ratkovic M, Covariate balancing propensity score. Journal of the Royal Statistical Society Statistical Methodology, Series B (2013), https://doi.org/10.1111/rssb.12027

Iacus SM et al., Causal Inference without Balance Checking: Coarsened Exact Matching, Cambridge University Press, (2017); https://www.cambridge.org/core/journals/political-analysis/article/abs/causal-inference-without-balance-checking-coarsened-exact-matching/5ABCF5B3FC3089A87FD59CECBB3465C0

Tchetgen EJT, Identification and estimation of survivor average causal effects, Statistics in Medicine, (2014); https://doi.org/10.1002/sim.6181

VanderWeele TJ and Ding P, Sensitivity Analysis in Observational Research: Introducing the E-Value, Annals of Internal Medicine (2017), https://doi.org/10.7326/M16-2607

Haneuse S *et al.*, Using the E-Value to Assess the Potential Effect of Unmeasured Confounding in Observational Studies. JAMA (2019);321(6):602–603. doi:10.1001/jama.2018.21554

Little RJA, A test of missing completely at random for multivariate data with missing values. Journal of the American Statistical Association (1988)

Ho D *et al.*, MatchIt: Nonparametric Preprocessing for Parametric Causal Inference, Journal of Statistcical Software, (2011), https://www.jstatsoft.org/article/view/v042i08

Mathur M. *et al.*, Web Site and R Package for Computing E-values, Epidemiology, (2018) doi: 10.1097/EDE.0000000000000864

Pishgar F *et al.,* MatchThem: Matching and Weighting after Multiple Imputation, The R Journal (2021)

Briggs A. *et al*., Decision modelling for health economic evaluation, Oxford University Press (2006)

7.2 DATA MANAGEMENT PLAN

PEACH Data Management plan v1.0 14.04.2021 FINAL.pdf

PEACH Metadata_v1.6_18.03.2022.xlsx

7.3 STUDY MASTER FILE AND STATISTICAL MASTER FILE

7.4 OTHER SOPS OR GUIDANCE DOCUMENTS

SAP DEVIATION LOG

**Document number:**		**Document version:**	
**Reason for deviation:**			
