# Supplementary material for: Procalcitonin Evaluation of Antibiotic Use in COVID-19 Hospitalised Patients (PEACH): Protocol for a Retrospective Observational Study
Source: Methods Protoc. 2022 Nov 28;5(6):95. doi: 10.3390/mps5060095 (PMC9786133; doi:10.3390/mps5060095)
Supplement: Supplementary file 1 [file mps-05-00095-s001.zip › mps-1985998-SI/Supplementary Table S1 05.10.22.pdf]

Procalcitonin Evaluation of COVID-19 Hospitalised patients (PEACH): protocol for a retrospective observational study.

Supplementary File

Table S1 –Variables to be collected for analysis.

**Variable**	**Measure**	**Reason**
Day 1	Date of COVID-19 diagnosis	To define reference point for analysis and to define 'baseline' (Day 1 +/- 1 day) for variable collection at time of diagnosis
Age	Age category:<br>• 16-49<br>• 50-59<br>• 60-69<br>• 70-79<br>• >80	Confounding factor, describe study population (Exploratory analysis)
Ethnicity	Category:<br>• White: Welsh/English/Scottish/Northern Irish/British<br>• White: Irish<br>• White: Gypsy or Irish Traveller<br>• White: Any other White background<br>• Mixed/Multiple ethnic groups: White and Black Caribbean<br>• Mixed/Multiple ethnic groups: White and Black African<br>• Mixed/Multiple ethnic groups: White and Asian<br>• Mixed/Multiple ethnic groups: Any other Mixed / Multiple ethnic background<br>• Asian/Asian British: Indian<br>• Asian/Asian British: Pakistani,<br>• Asian/Asian British: Bangladeshi<br>• Asian/Asian British: Chinese,<br>• Asian/Asian British: Any other Asian background<br>• Black / African / Caribbean / Black British: African<br>• Black / African / Caribbean / Black British: Caribbean	Baseline Characteristics

	• Black / African / Caribbean / Black British: Any other Black / African / Caribbean background<br>• Other ethnic group: Arab<br>• Any other ethnic group	
Sex	Male/female	Baseline Characteristics
Height and Weight	(i) Height and date measured<br>(ii) Weight and date measured	Baseline Characteristics
Comorbidity	(i) Quality Outcome Framework registered conditions<br>(ii) Primary Care Frailty Scores:<br>1 Very Fit<br>2 Well<br>3 Managing well<br>4 Vulnerable<br>5 Mildly Frail<br>6 Moderately Frail<br>7 Severely Frail<br>8 Very Severely Frail<br>9 Terminally Ill<br>(iii) Penicillin allergy or sensitivity status:<br>• Yes<br>• No<br>• Unknown	Confounding factors (Baseline Characteristics)
Smoking status	Category:<br>• No<br>• Yes<br>• Ex-Smoker<br>• Not Known	Baseline Characteristics
Index of multiple deprivation (IMD)	(i) IMD - Score<br>(ii) IMD - Rank<br>(iii) IMD – Decile	Confounding factor (Exploratory analysis)
Antibiotics/Antivirals used during treatment of episode	(i) Agent<br>(ii) Dose<br>(iii) Route<br>(iv) Frequency<br>(v) Start date<br>(vi) Stop date	Primary and secondary outcomes -<br>Used to derive: days and DDDs of 'early', 'late' and total antibiotic treatment.
Date of hospital admission/discharge	(i) Date of hospital admission and discharge<br>(ii) Date of ICU admission and discharge<br>**(iii) Date of death**	Secondary outcomes -<br>Used to derive: days - length of hospital stay, ICU length of stay, and mortality rates (Survival time).
Resuscitation status and level of care preference	Categories:<br>(i) Documented decision about resuscitation status:<br>• Yes/No<br>(ii) Documented decision regarding level of care:<br>• Yes/No	Confounding factor

	(iii) Decision in the event of a cardiorespiratory arrest:<br>• No (do not resuscitate)<br>• Yes (resuscitate)	
Presence and location of consolidation/ ground glass changes on lung imaging	(i) COVID-19 categorisation of imaging:<br>• X-ray uncoded<br>• Definite (CVCX1)<br>• Normal (CVCX0)<br>• Indefinite (CVCX2)<br>• Typical (non-COVID CVCX3)<br><br>(ii) Date of Baseline Imaging<br>(iii) Date of imaging where new/worsening consolidation identified	Confounder and secondary outcomes –<br>Dates used to derive: Time to new consolidation
Physiological observations at time of diagnosis (day 1 = Positive COVID-19 test date)	(i) Respiratory rate<br>(ii) Systolic blood pressure<br>(iii) Pulse rate<br>(iv) ACVPU<br>(v) Glasgow coma score<br>(vi) Temperature<br>(vii) Oxygen saturation<br>(viii) Oxygen requirement (was supplemental oxygen given?)	Confounding factor -<br>Used to derive:<br>(i) qSOFA<br>(ii) NEWS2 score<br>(iii) CURB-65 scores<br>(iv) 4C mortality score
Laboratory tests:	(i) Positive COVID-19 test date (day 1)<br><br>(ii) PCT test date	Primary Outcome -<br>Week of test date.<br><br>Secondary Outcome -<br>Study test, within 3 days of COVID test for inclusion in PCT group
Laboratory tests	At baseline (day 1 or -/+ 1 day COVID-19 positive test date):<br>• Urea<br>• C-reactive protein<br>• Troponin<br>• Ferritin<br>• D-dimer<br>• White cell count<br>• Lymphocyte count<br>• Neutrophil count<br>• Haemoglobin<br>• Platelets<br><br>Day 1, day 2 and day 3:<br>• Creatinine<br>• AKI stage	Confounding factors<br>(Exploratory analysis)
Laboratory tests	Microbiology at baseline (day 1 or -/+ 1 day COVID-19 positive test date) or	Secondary Outcomes -

	follow-up. Results and date of sampling for:<br>(i) Blood culture<br>(ii) Respiratory (Bronchoscopy/BAL cultures)<br>(iii) Sterile site culture	Used to derive resistant bacterial infection rates and time to event.
Laboratory tests	(i) GDH ELISA results<br>(ii) *C. difficile* toxin and toxin gene testing results<br>(iii) Presence of Respiratory virus or secondary viral infection	Secondary Outcomes
